# Artificial Intelligence for Histology-Based Detection of Microsatellite Instability and Prediction of Response to Immunotherapy in Colorectal Cancer

**DOI:** 10.3390/cancers13030391

**Published:** 2021-01-21

**Authors:** Lindsey A. Hildebrand, Colin J. Pierce, Michael Dennis, Munizay Paracha, Asaf Maoz

**Affiliations:** 1 Department of Medicine, Boston University School of Medicine and Boston Medical Center, Boston, MA 02118, USA; Lindsey.Hildebrand@bmc.org (L.A.H.); Colin.Pierce@bmc.org (C.J.P.); mjdennis@health.ucsd.edu (M.D.); mparacha@bu.edu (M.P.); 2 Division of Hematology Oncology, Department of Medicine, University of California San Diego, San Diego, CA 92093, USA; 3 Department of Medical Oncology, Dana-Farber Cancer Institute and Harvard Medical School, Boston, MA 02215, USA

**Keywords:** colorectal cancer, microsatellite instability, DNA mismatch repair, tumor immunology, immunotherapy, digital pathology, convolutional neural network, deep learning, machine learning, artificial intelligence

## Abstract

**Simple Summary:**

Defects in a DNA repair pathway called mismatch repair (MMR) can lead to cancer, including colorectal cancer (CRC). The detection of mismatch repair deficiency (dMMR) is based on molecular tests, one of which is microsatellite instability (MSI) testing. Detecting tumors with dMMR/MSI is important for the identification of patients with Lynch Syndrome and determining if patients may benefit from immunotherapy. Recently, artificial intelligence has been evaluated as a method to predict MSI/dMMR directly from tissue slides that are available for most cancer patients. We review the data regarding the utility of machine learning for dMMR/MSI classification, including its accuracy and limitations, focusing on CRC. We also provide an overview of previous efforts to predict MSI from tissue slides and background regarding the use of artificial intelligence for image analyses. We summarize recent efforts to use artificial intelligence for the prediction of MSI and discuss the implications for predicting response to immunotherapy.

**Abstract:**

Microsatellite instability (MSI) is a molecular marker of deficient DNA mismatch repair (dMMR) that is found in approximately 15% of colorectal cancer (CRC) patients. Testing all CRC patients for MSI/dMMR is recommended as screening for Lynch Syndrome and, more recently, to determine eligibility for immune checkpoint inhibitors in advanced disease. However, universal testing for MSI/dMMR has not been uniformly implemented because of cost and resource limitations. Artificial intelligence has been used to predict MSI/dMMR directly from hematoxylin and eosin (H&E) stained tissue slides. We review the emerging data regarding the utility of machine learning for MSI classification, focusing on CRC. We also provide the clinician with an introduction to image analysis with machine learning and convolutional neural networks. Machine learning can predict MSI/dMMR with high accuracy in high quality, curated datasets. Accuracy can be significantly decreased when applied to cohorts with different ethnic and/or clinical characteristics, or different tissue preparation protocols. Research is ongoing to determine the optimal machine learning methods for predicting MSI, which will need to be compared to current clinical practices, including next-generation sequencing. Predicting response to immunotherapy remains an unmet need.

## 1. Introduction

Colorectal cancer (CRC) is the third most common and second most deadly cancer worldwide, causing an estimated 880,000 deaths in 2018 [1]. Mortality rates for CRC have been declining in many countries due to improved screening efforts and therapeutic advances [2], but both the incidence and mortality of CRC have been increasing in patients under the age of 50 in high-income countries [3]. CRC is a heterogenous group of diseases (subtypes) with differences in epidemiology, anatomy, histology, genomics, transcriptomics and host immune response [4,5,6,7]. This heterogeneity leads to disparate clinical presentation, survival and response to therapy [8,9,10,11].

One of the clinically relevant subtypes of CRC is DNA mismatch repair deficient (dMMR) CRC. dMMR occurs due to pathogenic alterations in genes involved in the MMR system (*MLH1, MSH2/EPCAM, MSH6*, and *PMS2*) [12,13,14]. Several mechanisms can lead to dMMR, the most common being somatic hypermethylation of the *MLH1* gene promoter [14]. In patients with Lynch Syndrome, who carry a germline pathogenic mutation in one of the MMR genes, an additional somatic alteration can occur (“second hit”), leading to the phenotype of dMMR tumors. Sporadic bi-allelic somatic mutations in the MMR genes can also occur [13]. Deficiencies in MMR cause high rates of mutations throughout the DNA, especially in microsatellites, regions of DNA in which short sequences of nucleotides are repeated in tandem [12]. Thus, dMMR results in microsatellite instability (MSI), which is a highly sensitive marker of dMMR [13].

MSI has diagnostic, prognostic, and therapeutic implications in CRC and other cancers. The detection of dMMR/MSI is recommended as a screening test for Lynch syndrome for every case of CRC [14]. Confirmation requires germline testing and can inform surveillance and treatment decisions for both the patient and their relatives [15]. CRC patients with MSI generally have a better prognosis [13,16,17], which may be explained by a robust host immune response to tumor neoantigens [10,11,18]. Lastly, MSI status can inform treatment decisions, as patients with MSI tumors may be eligible for immune checkpoint inhibitor (ICI) therapy [19], and the benefit of fluorouracil-based chemotherapy regimens for tumors with MSI has been questioned [20,21,22,23]. 

**Figure 1:**
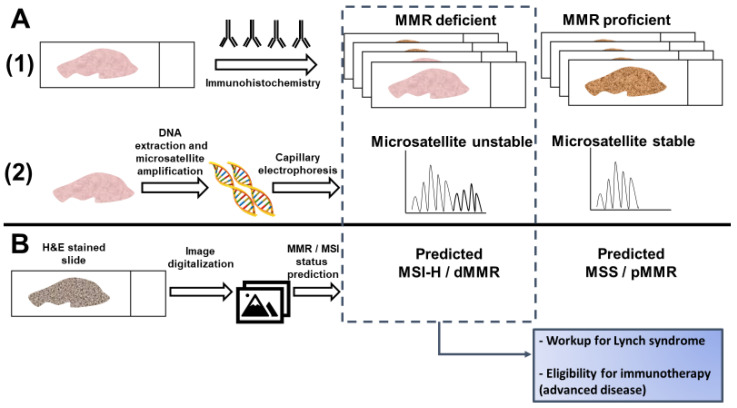
Detection of microsatellite instability (MSI) or mismatch repair (MMR) deficiency is performed by (**A1**) Immunohistochemistry of the mismatch repair proteins or (**A2**) PCR amplification of consensus microsatellite repeats that are analyzed with capillary electrophoresis. Inference of MSI/MMR status from next generation sequencing (NGS) is not presented. (**B**) MSI/MMR status can be predicted from hematoxylin and eosin (H&E) stained slides, without requiring molecular analyses (see Figure 2). Detection of MSI/dMMR has implications for Lynch Syndrome screening and determining eligibility for immune checkpoint blockade in advanced disease. MSS: microsatellite stable. MSI-H: high microsatellite instability. pMMR: proficient mismatch repair. dMMR: deficient mismatch repair.

Current testing for dMMR/MSI requires either an immunohistochemical analysis of MMR protein expression or a PCR-based assay of microsatellite markers [14]. While guidelines set forth by multiple professional societies recommend universal testing for dMMR/MSI [24], these methods require additional resources and are not available at all medical facilities, so many CRC patients are not currently tested [25]. Recently, artificial intelligence has been evaluated as a method to predict MSI directly from hematoxylin and eosin (H&E) stained slides (Figure 1). If successful, this approach could have significant benefits, including reducing cost and resource-utilization and increasing the proportion of CRC patients that are tested for MSI. 

We review the emerging data regarding the utility of artificial intelligence for MSI classification, focusing on CRC. We provide (1) an overview of pathologic predictors of MSI, (2) a background regarding the use of artificial intelligence for image analyses, (3) a summary of recent efforts to use artificial intelligence for the prediction of MSI, and (4) a discussion about the implications for predicting response to immunotherapy.

## 2. Histological and Clinical Predictors of Microsatellite Instability

With the significant cost and non-universal availability of the molecular testing required to determine MMR/MSI status, studies have sought to predict MSI based on routinely available data, such as clinical information and histopathology [26]. CRC tumors with MSI are associated with certain histological features, detectable via standard H&E staining, and clinical data, such as patient age and tumor location [26,27,28]. Similar observations have been made in other tumors enriched for MSI, such as endometrial cancer [29]. These associations may present a means of identifying the tumors most likely to have the dMMR phenotype, and therefore the patients most likely to benefit from additional testing. They may also help to identify those at low risk who would be less likely to benefit. The targeted deployment of MMR/MSI testing could reduce costs and save resources [26]. Inferring MSI status may be considered in settings where MSI testing is not performed but is unlikely to be adopted in resource-rich settings unless the prediction accuracy is near-perfect. 

Several clinicopathologic predictors of MSI have been discovered and several groups have proposed models for MSI prediction (Table 1). Histological features such as signet ring cells, mucinous or medullary morphology, and poor differentiation are significantly associated with MSI status, but show poor sensitivity for MSI prediction on their own [27,30]. Correlations between MSI and immunological features of tumor pathology, such as measurements of tumor infiltrating lymphocytes (TILs) [11,26,28,31] and specific histological structures such as the Crohn’s-like lymphoid reaction (CLR), are well established in the literature [18,26,28]. CLR represents CRC-specific tertiary lymphoid aggregates [18]. The host response to MSI tumors is attributed to the high tumor mutational burden (TMB) and the abundance of immunogenic mutations, including insertion-deletion mutations, but other factors may contribute [32,33,34]. The Revised Bethesda Guidelines for MSI testing in CRC suggested testing tumors with “MSI histology” in patients younger than 60 years of age [35]. MSI histology was defined as the presence of TILs, CLR, mucinous/signet-ring differentiation, or medullary growth pattern. One of the histopathological features most strongly associated with MSI is the density of TILs [26,27,30]. When TIL density was assessed as a potential predictor of MSI, the area under the receiver operating characteristic curve (AUC) was 0.73. With a cutoff value of 40 lymphocytes/0.94 mm^2^, MSI status could be predicted with a sensitivity of 75% and a specificity of 67% [30]. However, given that TIL density can vary across tumor area, this study using surgical specimens likely yielded a greater AUC than would be achieved with smaller biopsy specimens, such as those typically available from sites of metastasis.

Multiple histological and clinical variables have been incorporated into algorithms designed to predict MSI status. The MsPath score was developed to predict MSI in patients under the age of 60 [27]. Using a scoring system incorporating age, anatomical site of the primary tumor, histologic type, tumor grade, and the presence or absence of TILs and CLR, an AUC of 0.89 was achieved when the model was tested against a separate validation cohort (Table 1). Validation of the MsPath score in a population based-cohort showed that its accuracy was insufficient for the selection of patients for Lynch Syndrome germline testing, misclassifying 18% (2/11) of patients with a pathogenic mutation in *MLH1/MSH2* [39]. Another scoring scheme by Greenson et al. incorporated similar variables but included lack of dirty necrosis in the model and was derived from a population that included patients of all ages [26]. The features associated with MSI all had a negative predictive value >90%. This model yielded an AUC of 0.85 based on the study cohort alone (no validation cohort was tested) (Table 1). Over half of the tumors analyzed had less than 5% chance of harboring MSI, presenting the potential for significant cost savings [26]. In another cohort, the model by Greenson et al. detected 93% of tumors with MSI and outperformed MsPath [40]. 

The PREDICT score was developed to improve on MsPath and other models [36]. It included variables that were significantly associated with MSI in a multivariable regression model, including age <50, right sided location, TILs, a peritumoral lymphocytic reaction, any mucinous component and increased stromal plasma cells [36]. PREDICT reported a sensitivity of 97% for the detection of MSI with an AUC of 0.924 in the validation cohort (Table 1). The RERtest6 model was developed to maximize the negative predictive value and included tumor location, growth pattern, solid and mucinous pattern, TIL and CLR [38]. The model had an accuracy of 92% in the global cohort and a negative predictive value of 97.9% (Table 1). The prevalence of MSI was 8.5% in this study. If this model were applied as screening for MSI in this study population, only 10% of patients would need confirmatory testing [38].

Another large study of MSI prediction from commonly available clinico-pathologic data included over three thousand patients over 50 years of age in Japan [37]. Female sex, proximal location, tumor size larger than 60 mm, mucinous component and *BRAF* mutation were associated with MSI and were included in a composite score used for prediction. CLR and TILs were not evaluated. In the validation cohort, the AUC was 0.856. Patients with *MLH1* promoter hypermethylation had higher scores than patients with Lynch Syndrome, as a result of the known association between *BRAF* mutations and *MLH1* hypermethylation and the high score given to *BRAF* mutations in the model. Overall, the performance of the model was disappointing, with approximately 25% of MSI tumors misclassified at the proposed threshold [37].

The encouraging performance of certain histology-based prediction models has not been sufficient to supersede universal testing for MSI/dMMR. Measurement of the variables for MSI prediction requires significant effort and expertise by pathologists, and inter-rater differences may affect the perceived reliability of histology-based scoring systems [41,42]. However, this work is fundamental to the premise that MSI can be predicted from histology, which has now been proposed as a task for deep learning from digital pathology [43] (Figure 1).

## 3. What Is Deep Learning and How Does It Apply to Digital Pathology? 

*Artificial intelligence* is a broad term that characterizes the ability of machines to mimic intelligent human actions. *Machine learning* is a subset of artificial intelligence that allows computer systems to improve their performance (“learn”) without being explicitly programmed [44]. *Deep learning* is a branch of machine learning that incorporates several layers of computational operation for the execution of complex tasks [45]. In the context of computer vision, deep learning often utilizes *convolutional neural networks* (CNNs) [44,46,47]. CNNs are designed to process raw data in the form of multiple arrays, such as color images; their structure is inspired by architecture found in the human brain’s visual cortex [45]. To achieve their goals of classifying images (e.g., is this a tumor or not?) or identifying objects, CNNs are trained on datasets that have been labeled with the desired output [41,44].

**Figure 2:**
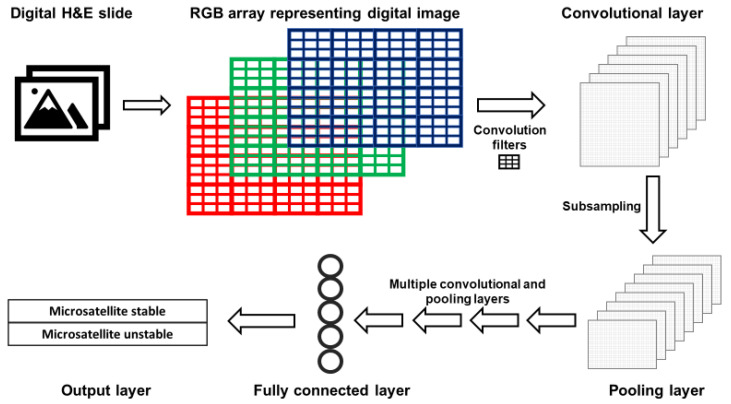
Simplified architecture of a convolutional neural network (CNN). Images acquired with digital pathology are composed of pixels. The color of each pixel can be represented with values of the red, green and blue (RGB) scheme. RGB arrays are subjected to filters to create a convolutional layer. Filters detect specific features from an image (e.g., lines, edges). A pooling layer with a reduced number of parameters is created by summarizing (subsampling) the input of the convolutional layer. After a defined number of convolutional and pooling layers, fully connected layers are created. Fully connected layers are uni-dimensional layers from which the output is predicted.

The layers of CNNs are arranged such that deeper layers represent increasingly synthesized features of an image. For example, the first layer typically detects edges; the second represents motifs related to the arrangement of edges; subsequent layers combine motifs into a representation of objects, and so on [45]. A simplified version of a CNN is presented in Figure 2. Images are represented as red, green and blue (RGB) color arrays such that each pixel in the image is represented by three numbers. RGB arrays are then subjected to filters. Filters are matrices that are used to learn specific features that are not prespecified but will help the CNN perform its task. For example, first layer filters often detect object edges in different orientations. This happens in the following fashion. The RGB arrays are convolved (a mathematical operation) with filters to create a multi-dimensional convolutional layer (Figure 2). To mimic the physiological “firing” of a neuron, a non-linear activation function is applied to the results of the convolution operation. Next, a pooling, or subsampling, procedure can be used to summarize the features from the convolutional layer and reduce the number of parameters, such that a pooling layer is created (Figure 2). 

After a series of convolutional and pooling layers, fully connected layers are created. A fully connected layer is typically a unidimensional layer that is used to create the output prediction function (Figure 2). When the CNN output is generated, it is compared to the “true” label assigned to the data. When the CNN output is wrong, the CNN modifies its filters to improve the prediction accuracy of the CNN, thus learning the features associated with the desired output. 

Training well-performing CNNs requires large datasets for training, testing and validation [47,48]. For the purpose of supervised learning such as image classification, these data need to be labeled according to the desired output. Many of the CNNs used for deep learning from digital pathology were originally developed for object detection and image classification as part of the ImageNet challenge [47,49,50,51]. ImageNet is an annotated database of over a million non-medical images for which increasingly efficient CNNs were designed. These CNNs are robust to diverse classification and image recognition tasks and have been applied to digital pathology tasks [52]. Another advantage of applying CNNs to new tasks is the ability to use *transfer learning*, building on previously trained CNNs to perform a new task. Computationally, this means that not all layers of the CNN need to be trained again, and adequate performance can be achieved using a smaller dataset. 

The availability of digital pathology datasets, annotated with clinical and molecular data, has led to a growing number of studies to evaluate the performance of CNNs on digitalized histology slides [41,46,52]. Such datasets include The Cancer Genome Atlas (TCGA) and the Genotype-Tissue Expression (GTEx) project. The tasks that have been assigned to CNNs are diverse, including predicting clinical outcome and response to therapy, identifying molecular features, and others [52]. While CNNs have similar features, as described above, they differ from one another in their architecture, including the size, sequence and number of layers and filters, their number of parameters, and the connections between the layers of the CNN. As a result, CNNs vary in their computational efficiency and performance [52]. Common CNNs for the purpose of image classification include VGG [49], ResNet [50] and Inception [51].

## 4. Application of Deep Learning to Digital Pathology in Oncology

Deep learning is being explored for a myriad of research and clinical uses. Emerging studies in oncology have suggested that deep learning can be used to predict the diagnosis, prognosis, and response to treatment using histopathology digital slides as input [41]. For example, a deep learning algorithm has been developed for the prediction of the histology-based Gleason score in prostate cancer [53]. The deep learning algorithm outperformed general pathologists, but accuracy in assigning Gleason scores was only 0.70 when compared with reference scores provided by genitourinary pathologists [53]. In CRC, a deep learning method predicted five-year CRC-specific survival from spot images of H&E tumor slides, independent of tumor stage and grade [54]. MSI and the immune response to the tumor were not reported, and pathologists were provided only with spot images for risk stratification. Although conceptually intriguing, the comparison with pathologist performance in this study does not reflect standard pathologic evaluation. A “deep stroma score” has also been generated by using CNN transfer learning for the prediction of survival in CRC [55]. 

Recently, ten CNNs were used to generate an independent prognostic biomarker for CRC-specific survival, termed “DoMore-v1-CRC” [56]. This biomarker was associated with several clinical and molecular features and was trained on different resolutions of H&E images. The immune response to tumors was not reported, and the biomarker was not compared with the Immunoscore [57], a validated prognostic marker measuring immune cells in the CRC microenvironment using digital pathology. A prospective trial of tailoring therapy to prognostic subgroups is planned based on these results [56].

Deep learning has also been used to predict tumor molecular features, such as genomics, transcriptomics and proteomics. Using an Inception CNN, researchers classified non-small cell lung cancer into histological subtypes and predicted the mutational status of several genes in lung adenocarcinoma [58]. Histological subtype classification achieved high AUC (0.97) when trained on TCGA data, but performance was worse for independent datasets, requiring manual identification of tumor areas by pathologists. Some of the misclassifications included the labeling of blood vessels, clots, inflammation, and necrosis as lung adenocarcinoma and the labeling of cartilage as lung squamous cell carcinoma. For the purpose of genomic prediction, mutations in *STK11, EGFR, KRAS*, *TP53* and other genes were predicted with AUCs of 0.733 to 0.856. 

Using the ResNet CNN, the prediction of PD-L1 expression was performed from H&E slides in non-small cell lung cancer [59]. Prediction was good for adenocarcinomas (AUC = 0.83) but did not perform well for squamous cell histology (AUC = 0.64). This difference highlights potential challenges in the generalizability of such CNNs to different histological subtypes and different tumor sites, and how the composition of the training dataset may influence CNN performance. In breast cancer, the VGG CNN was used to predict tumor grade, estrogen receptor status, histological subtype, and RNA-based molecular subtype and recurrence risk score [60]. Accuracy was highest for the task of histological subtype classification, raising the possibility that output based on visual patterns may be more amenable to prediction than transcriptomic data. Consensus molecular subtypes of CRC, derived from transcriptomic data, have also been predicted from histology using an Inception CNN [61]. This study used three datasets and suggested that the CNN learned features that are specific to the dataset, thus potentially biasing the learning process and limiting generalizability. To overcome this, the authors implemented adversarial training to minimize the weight of dataset-specific features. Histological predictors of the consensus molecular subtype 1 were underrepresented in a dataset comprised of rectal cancer biopsies, requiring adjustment of the classification probabilities. 

These examples demonstrate the broad applications of machine learning methods to digital pathology in oncology as well as some of the caveats to their performance. Careful evaluation of individual studies is required, as methods differ considerably and at times include modifications that may hamper the feasibility or generalizability of the methods proposed. Most studies are retrospective and have not been evaluated in prospective clinical trials with standard of care comparator methods. In addition, CNNs learn features that are biased and do not reflect biological differences. This can be partially mitigated if features that may cause bias are known, but since the features determining CNN output are often unknown, validation in additional cohorts is crucial. 

## 5. Predicting MSI Status with Deep Learning 

Recently, several studies have investigated the potential for CNNs to predict MSI from H&E stained histological samples. Kather et al. trained and tested CNNs on gastric, endometrial, and colorectal samples that were snap-frozen or formalin-fixed paraffin-embedded (FFPE) [43]. FFPE slides are routinely used for histological diagnosis and immunohistochemistry. Fixation with formalin and embedding with paraffin are performed to maintain tissue architecture and morphology, and to allow long-term preservation at room temperature. The process of generating an FFPE slide requires many hours and the fixation process results in the cross-linking of DNA and proteins that can impair the performance of molecular analyses. Snap-frozen tissue is not routinely obtained but can be used for intraoperative diagnoses because it can be rapidly reviewed by a pathologist. Snap-frozen tissue can also be used for extensive molecular analyses [62,63]. The morphological quality of snap-frozen tissue is not considered sufficient to render a definitive diagnosis, and confirmation using FFPE slides is typically required [64,65,66]. All CNNs in the study by Kather et al. had been pretrained on the ImageNet database, and only the last ten layers of the CNNs were trainable. After assessing the performance of five CNNs in differentiating tumor tissue from healthy tissue, the CNN ResNet-18 (a ResNet with 18 layers) was selected for further evaluation based on its strong performance and smaller number of parameters. The advantage of a CNN with a smaller number of parameters is a decreased risk of overfitting the data and increased likelihood of maintaining performance when applied to a validation cohort. ResNet-18 was trained with two sets of CRC (fresh frozen and FFPE slides) and one gastric cancer dataset (FFPE) from TCGA (Table 2). Tumor tissue was divided into smaller tiles, each of which was separately analyzed and assigned a predicted MSI score. Predicted MSI status for each slide was determined by the predicted MSI status of the majority of its constituent tiles. 

Using this process, the CNN was able to detect MSI in snap-frozen and FFPE TCGA samples with similar AUCs to those achieved with previous pathology-based scoring systems such as MsPath and the model by Greenson et al. (0.84 for snap-frozen CRC samples, 0.77 for FFPE CRC samples, and 0.81 in FFPE gastric adenocarcinoma samples). This level of performance was maintained when the CNN trained on FFPE CRC samples was tested on an external validation cohort from the DACHS (Darmkrebs: Chancen der Verhütung durch Screening) study (Table 2), which consisted of FFPE CRC samples from Germany (AUC 0.84). The authors also tested the classification performance of the ResNet when applied to slides with limited tissue, finding that performance plateaued with a quantity of tissue that is available from standard needle biopsies [43].

To attempt to identify what pathological features the ResNet used to make its classifications, tumor regions that were assigned high or low MSI scores were visually inspected. Areas predicted by the CNN to represent MSI often showed characteristics consistent with known pathological correlates of MSI, such as poor differentiation and lymphocytic infiltration. PD-L1 expression and an interferon gamma transcriptomic signature were correlated with the proportion of a sample’s tiles predicted to have MSI. This finding is consistent with previous data showing high expression of PD-L1 and interferon gamma in CRC with MSI [73,74]. 

Despite encouraging performance for MSI classification in similar cohorts, testing against different cohorts revealed some limitations. CNNs trained on snap-frozen CRC samples or gastric adenocarcinoma samples did not perform as well as the CNN both trained and tested on FFPE CRC samples. When the CNN was trained to detect MSI in endometrial cancers, its performance was significantly reduced to an AUC of 0.75, raising the possibility that the CNN is learning tissue-specific features associated with MSI. Additionally, the CNN trained on TCGA gastric adenocarcinomas did not perform as well when tested on a Japanese gastric adenocarcinoma cohort (AUC 0.69), possibly due to distinctive histological patterns seen in gastric adenocarcinomas in this cohort [43]. 

Other studies have attempted to improve upon these results using other CNNs and machine learning techniques (Table 2). In a follow up study by Kather et al., the prediction of MSI was performed as a benchmark task by various CNNs, which were pretrained on the ImageNet database [67]. The ResNet and Inception CNNs were outperformed by the DenseNet [75] and ShuffleNet [76] architectures. ShuffleNet, a CNN optimized for mobile devices, was able to achieve an AUC of 0.89 when trained on a CRC cohort from TCGA and validated on the DACHS CRC cohort (Table 2). The ResNet used for the previous study by Kather et al. achieved an AUC of 0.84 [43,67]. 

Another group reports improvement upon the results by Kather et al. in terms of overall predictive accuracy and generalizability to different cohorts [68]. This study also used ResNet-18 to assign each tile within the tumor area an MSI likelihood. However, multiple instance learning was used to train the CNN to classify the whole slide image. Multiple instance learning assumes that not all tumor regions contribute the same amount of information to the task of classification of the tumor as a whole [77]. Certain regions or patterns found in limited areas of a sample may be more important to determining the likelihood of the tumor being MSI. For example, any mucinous differentiation increases the likelihood of a tumor harboring dMMR/MSI [26,28]; this may be focal and not seen in the majority of tumor areas. Two different multiple instance learning methods were used in this study, and their input was integrated into a final ensemble predictor (Table 2). This ensemble classifier achieved an AUC of 0.885 [68], which was better than the performance reported by Kather et al. [43].

This group also found a significant reduction in AUC (0.650) when the TCGA-trained ensemble classifier was tested on a cohort of Asian patients with samples acquired with a different slide preparation protocol [68]. They were able to overcome this reduction in performance by transfer learning. By adding increasing proportions of data from the Asian cohort to the training set, they were able to achieve an AUC of 0.850 with 10% samples from the Asian cohort, with continued improvement up to an AUC of 0.926 with 70% samples from the Asian cohort (Table 2) [68]. Pathologic signatures were derived from the model and were associated with known features of MSI, including TMB and insertion-deletion mutational burden, as well as transcription signatures of immune activation. 

A conference paper by Wang et al. also assessed an alternative technique, Patch Likelihood Histogram (PALHI), for integrating tile-level MSI predictions into patient-level predictions using whole slide images from a TCGA endometrial cancer cohort [78]. First, a ResNet-18 pre-trained on ImageNet was trained to predict MSI for individual tiles on a subset of the TCGA cohort. PALHI then generated a histogram of the patch-level estimated MSI likelihoods, which were used to train a machine learning classifier called XGBoost to make patient-level predictions. The performance of a pipeline using PALHI to make patient-level predictions was compared to pipelines using another machine learning method, Bag of Words (BoW) and the “majority voting” method, using another subset of the TCGA cohort as a testing set. The three methods were each trained on both patches assigned binary “hard labels” and patches assigned “soft labels,” or MSI probabilities. The PALHI method trained using “soft labels” yielded the best performance on the test set, with an AUC of 0.75. By comparison, the AUCs for BoW and the majority method using “soft labels” were 0.71 and 0.56, respectively [78].

Transcriptomic prediction from H&E slides has also been used to improve MSI prediction when limited training data are available [69]. First, features were extracted from each tissue tile using the ResNet-50, pretrained on the ImageNet database. These features served as the input for a custom multilayer perceptron, which was trained to predict gene expression from RNA-Seq data. *Multilayer perceptrons* are neural networks composed of fully connected layers, typically without convolutional layers. This neural network was trained on pan-cancer and tissue-specific TCGA datasets and was able to predict several expression signatures, including adaptive immune response signatures [69]. For MSI prediction, the authors simulated a situation where a limited number of training slides are available at two sites. They showed that, using the transcriptomic representation trained at one site, they could improve MSI prediction at the second site. However, when increasing proportions of data at the second site were used for MSI prediction without integrating transcriptomic representation, this advantage was largely lost. Neither method achieved an AUC > 0.85 and no external validation set was used (Table 2) [69]. It is unclear if this approach would be applicable in real-life settings. 

In a conference paper submitted to the 1st Conference on Medical Imaging with Deep Learning (MIDL 2018) [70] and a related patent [79], adversarial learning was used to improve the generalizability of CNNs for MSI prediction across different cancers. The Inception-V3, ResNet-50 and VGG-19 CNNs were compared; Inception-V3 was chosen for downstream analysis. TCGA samples were used for both testing and training; this study did not use an external validation dataset. MSI status was categorized as stable, low instability or high instability. Inception-V3 was trained on CRC samples and achieved a slide-level accuracy of 98.3% with an internal validation set of 10% of TCGA slides. It is unclear if this level of accuracy represents overfitting. Accuracy was poor when applied to endometrial carcinoma samples at 54%, whereas training the CNN on both CRC and endometrial carcinoma decreased the accuracy of MSI prediction for CRC to 72% (Table 2). This CNN also performed poorly at classifying MSI in gastric adenocarcinoma with a slide-level accuracy of 35%. Next, a tumor type classifier was added to the CNN with an adversarial objective—to decrease the ability of the model to predict tumor type. The rationale for creating this adversarial objective is to remove tissue-specific features that are learned by the CNN, such that the model will recognize the features associated with MSI better. Adversarial training improved MSI classification across the three cancer types, but accuracy remained poor for gastric adenocarcinoma at 57% [70]. 

Focusing on endometrial cancer, a recent study available as a preprint generated CNNs that had three branches of an InceptionResNet architecture, each analyzing tiles at a different resolution [71]. An optional fully connected layer incorporating clinical features was also evaluated as a fourth branch. This structure, termed Panoptes, allowed the model to take into account both tissue-level and cellular-level structures, as would a human pathologist using a microscope. MSI classification was one of several tasks that the CNNs were trained to do. While the complex architecture showed strong performance in predicting many histological and molecular features, MSI was best predicted by the existing InceptionResnetV1 architecture, with an AUC of 0.827 (Table 2), which outperformed Kather’s previously described ResNet-18 architecture (AUC 0.75). The inclusion of clinical data did not seem to improve the model’s performance: when the age and BMI of the patient were added into the model, its performance did not significantly improve [71]. Predicted MSI was correlated with certain histological features, including intratumoral and peritumoral lymphocytic infiltrates. 

The strongest-performing model for MSI prediction was developed by Echle et al. by training a CNN on a large cohort of H&E-stained CRC samples from the MSIDETECT consortium, which is comprised of whole slide images from TCGA, DACHS, the United Kingdom-based Quick and Simple and Reliable trial (QUASAR), and the Netherlands Cohort Study (NLCS) [72]. A modified version of the CNN ShuffleNet that was pre-trained on ImageNet was trained on whole slide images from MSIDETECT with known MSI or dMMR status and externally validated on a separate population-based cohort, Yorkshire Cancer Research Bowel Cancer Improvement Programme (YCR-BCIP). For each slide, tumor tissue was manually outlined and the slide was divided into smaller tiles. The patient-level prediction of MSI/dMMR was based on the average tile-level prediction for each patient. The CNN was first trained and tested on individual sub-cohorts. As in earlier-described studies [43,68,70], when a CNN trained on a single sub-cohort was tested on another sub-cohort, performance usually suffered. A positive correlation between the size of the training cohort and the performance of the model was noted. The CNN was then trained on increasing numbers of patients randomly selected from the MSIDETECT cohort. The model showed better performance with greater numbers of patients up until about 5000 patients, after which performance plateaued. After training with samples from 5500 patients, the model attained an AUC of 0.92 when tested on a separate set of patients from MSIDETECT. When tested on the external validation cohort (YCR-BCIP), the model attained a similarly impressive AUC of 0.95. Additionally, when slides were subjected to color normalization, the specificity at given levels of sensitivity increased and a slight improvement in AUC to 0.96 was demonstrated [72]. Though these results are encouraging, it is worth noting that the samples used to train and test this model were derived mostly from European patients. Further validation with more diverse cohorts and prospective studies will be necessary before this model can be applied in a broad clinical context. 

Subgroup analysis did reveal some variation in the model’s performance for certain tumor characteristics. While the performance was consistent for tumors at stages I-III (AUCs 0.91–0.93), the AUC for stage IV tumors was lower (0.83). The authors do not discuss potential explanations for this discrepancy, but there was a similar reduction in AUC for tumors with high histologic grade (AUC for high grade tumors was 0.83). The relatively low prevalence of MSI/dMMR in stage 4 colorectal cancers would have decreased the number of available images from this subgroup available for training, as would the fact that stage 4 tumors are more likely to come from biopsy specimens than complete resection samples. This lower performance for stage 4 tumors is unfortunate given that ICI therapy is currently primarily used in late-stage colorectal cancer, reducing the model’s potential utility for guiding treatment decisions. Additionally, the model predicted MSI more effectively for colon cancer (AUC 0.91) than for rectal cancer (AUC 0.83). Performance did not vary significantly by tumor molecular characteristics (e.g., mutation status) [72]. 

As noted above, a previous study demonstrated that the performance of ResNet-18 in classifying MSI status plateaued with a quantity of tissue that can be obtained by needle biopsy [43]. However, Echle et al. found a significant decrease in AUC when the CNN trained on surgical specimens was tested on YCR-BCIP biopsy specimens as compared to YCR-BCP surgical specimens (0.78 vs. 0.96). Though size of specimen may be a factor here, artifacts from specimen acquisition and the fact that samples were derived only from luminal tumor tissue may also affect performance. When the authors performed a 3-fold cross-validated experiment using YCR-BCIP biopsy specimens to both train and test, the AUC improved to 0.89 [72]. However, the model was not tested on samples from sites of metastasis, which are commonly biopsied in the clinical setting. Thus, machine learning models may be effective in classifying the MSI status of biopsy specimens, but will likely perform best when trained on similarly derived specimens. 

Taken together, these studies demonstrate that multiple CNNs and machine learning techniques are being evaluated for MSI prediction from histology. There is no clear consensus regarding the optimal network architecture. The use of large and diverse datasets for training may overcome some of the limitations of models whose classification accuracy for MSI status is worse when applied to datasets with differing characteristics, which could be the case when applying these methods across different health systems, regions and populations. With continued experimentation, improvement, and validation of existing models, the use of machine learning to predict MSI may reach a level of accuracy sufficient for clinical application in the future.

## 6. Predicting Response to Immunotherapy with Deep Learning

While MSI status is currently used to determine a CRC patient’s eligibility for ICIs, it is far from a perfect predictor of the efficacy of these treatments. Only 30–50% of CRC patients with MSI respond to ICIs. There is also a subset of microsatellite stable CRC that responds to ICI [32,80,81], demonstrating that ICIs could have a role in the treatment of early-stage proficient MMR tumors. Patients who receive these treatments are at risk for immune-related adverse events including thyroid dysfunction, hepatitis, colitis, pneumonitis and others [82]; there is increasing evidence of an association between response to therapy and the development of immune-related adverse events [83,84]. Thus, alternative methods of determining eligibility and predicting the efficacy and toxicity of ICIs are needed. 

Despite the potential for the prediction of MSI from histology discussed above, there is little published research using this method to predict ICI response, and to our knowledge, there are no published results concerning ICI response prediction in CRC. Machine learning can be used to predict ICI response in other tumors from various types of input data, including H&E staining, which may lay the groundwork for similar research in CRC. One study available in abstract form predicted ICI response in melanoma from pre-treatment H&E slides in patients who were treated with first-line ICI therapy [85]. A CNN was trained to classify slides into responders and non-responders and into those who experienced severe adverse events and those who experienced none. The model performed modestly well in predicting ICI response, despite training on slides from only 124 patients. The model was much less effective at predicting adverse events, and research incorporating immunologic biomarkers into the algorithm is ongoing [85]. A similar study on non-small cell lung cancer (NSCLC) samples used the spatial arrangement of TILs as detected by computer algorithms to train a machine learning classifier to predict response to nivolumab, achieving an AUC of 0.64 on an external validation cohort [86]. 

A variety of biomarkers have been evaluated for predicting response to immunotherapy, many of which can be predicted from histology leveraging deep learning. One such biomarker is TMB, which is associated with specific CNN-derived pathological signatures [68]. In most cancers, including CRC, TMB is associated with improved overall survival after treatment with ICIs [87,88]. This association is attributed to the heightened immune response elicited by the multitude of tumor neoantigens [89,90,91]. However, tumors with low TMB can respond to ICIs, as such tumors may harbor one or more highly immunogenic mutations. This was demonstrated in a case study of a patient with pembrolizumab-responsive proficient MMR metastatic CRC, who was found to have T-cell responses to at least one neoantigen expressed within their tumor [92]. In addition, a recent study of neoadjuvant ICI treatment showed that there was no difference in pretreatment TMB between early-stage proficient MMR (pMMR) CRCs that responded and those that did not [81]. MMR deficient CRC is substantially more immunogenic than unselected MMR proficient CRC [10,32], in part due to the high TMB including frameshift insertion-deletion mutations [34].

T cell infiltration in the tumor microenvironment has also been studied as a potential biomarker for ICI response. A recent study of early-stage CRCs including both proficient and deficient MMR tumors showed that neoadjuvant therapy with a combination of nivolumab and ipilimumab elicited a pathological response in all dMMR tumors and 27% of proficient MMR tumors [81]. Proficient MMR tumors that responded to this treatment could be predicted by the presence of TILs that co-expressed CD8 and PD-1 on pre-treatment biopsies. No other biomarkers were found to differ significantly between the proficient MMR tumors that responded and those that did not [81]. Increasing density of CLR was observed after treatment. Other factors likely play a role in the response of proficient MMR tumors to ICIs. For example, high expression of IL-17 has been suggested to abrogate the ICI-responsiveness of such tumors, even in the presence of TILs expressing CD8 and PD-1 [93]. CNNs have been used to predict the spatial organization and subtypes of T cells within the tumor microenvironment of CRC and other tumors [69,94,95]. These features have already been used to predict ICI response in NSCLC with some success [86].

CNNs have also successfully predicted PD-L1 expression [59], another biomarker that has been assessed as a potential predictor of ICI response. However, while mechanistically compelling and potentially predictive of ICI response in NSCLC, PD-L1 does not seem to be useful on its own in determining which CRC will respond to ICI [96]. PD-L1 expression was not associated with progression-free or overall survival in CRC patients treated with ICIs, and there was no significant difference between responders and non-responders in PD-L1 expression in pre-treatment samples from patients with dMMR metastatic CRC [32,97]

Other researchers have used different types of clinical data to train machine learning models to predict ICI response. For example, a machine learning method called ImmuCellAI was developed to predict the relative abundance of various types of T cells in pre-treatment samples of melanomas from gene expression data. They then developed a separate model to predict immunotherapy response based on these results, achieving an AUC of 0.80–0.91 [98]. The successful implementation of a similar machine learning technique involving ICI response prediction based on the expression of immune-related genes was also reported in NSCLC and triple-negative breast cancer [99,100]. 

Radiomics-based machine learning has also been used to predict response to immunotherapy in melanoma and NSCLC based on a defined set of features extracted from pre-treatment CT imaging of primary and metastatic tumors from patients treated with anti-PD-1 therapy [101]. The model produced a radiomic biomarker score for each lesion evaluated, from which anti-PD-1 response was predicted. By combining the predictions from each of an individual patient’s lesions, a patient level prediction of anti-PD-1 response could be made. The model achieved significant performance for both tumor types (AUC 0.76 for NSCLC and 0.77 for melanoma) [101]. A similar study used deep learning to develop a TMB radiomic biomarker that can divide NSCLC tumors into high- and low-TMB groups (AUC 0.81) and divide ICI-treated NSCLC patients into high and low risk groups with significantly different overall and progression-free survival [50]. Another model can predict the transcriptomic-based abundance of CD8 T-cells, and response to immunotherapy, from radiologic data. The resultant biomarker was found to be positively associated with response to treatment with anti-PD-1 and anti-PD-L1 therapies [102]. Fluorodeoxyglucose (FDG)-positron emission tomography (PET) scans have also been used to train deep learning networks to determine a biomarker quantifying CD8+ T cell activity against the tumor that can differentiate between those patients more likely to respond to immunotherapy and those who are less likely to respond [103].

Specific somatic mutations may affect tumor response to ICIs. Previous success in predicting genomic data from histology [58,60,71] suggests that it would be possible to perform for other genes, but validation for individual genes would be required. For example, mutations in the DNA polymerase epsilon (*POLE*) gene, which codes for an enzyme involved in DNA proofreading during replication, can lead to a very high mutational burden without MSI [104]. The predicted number of neoantigens produced by affected CRCs can significantly exceed that of CRCs with MSI [105]. CRCs harboring *POLE* mutations have quantities of TILs similar to those found in dMMR CRCs [106], demonstrating an adaptive host response to these tumors. At least one case report describes a robust response to pembrolizumab treatment in a patient with a metastatic, treatment-refractory microsatellite stable CRC with a confirmed *POLE* mutation [107]. Clinical trials are ongoing to determine the extent of the benefit of ICI treatment in CRC with *POLE* mutations [108]. However, the impact of these studies will be limited, as *POLE* mutations are only found in about 1–2% of CRCs [106]. 

While multiple machine learning methods have been used to predict ICI efficacy in tumors other than CRC, and several biomarkers associated with ICI efficacy in CRC have been identified, data are lacking regarding machine learning for the prediction of ICI efficacy in CRC. Optimal ICI response prediction in CRC and other cancers will likely require larger datasets and the integration of multiple types of biomarkers incorporating genetic, immunologic, and other data. Leveraging machine learning to predict ICI response in CRC is an appealing goal given the lack of a sufficiently accurate predictive biomarker. The prediction of ICI response from ubiquitously available clinical data, such as H&E slides and radiographic studies, could greatly improve access to these therapies.

## 7. Future Directions

Table 3 summarizes the potential advantages, current limitations, and suggestions for future development of machine learning for MSI classification from digital pathology. The potential to predict multiomic data from a universally available clinical specimen has substantial advantages that rely on the ability to achieve excellent classification with CNNs. Data that are not routinely collected (e.g., transcriptomics) can be predicted utilizing relatively limited resources if a digital pathology infrastructure already exists. With the increasing use of omics data for clinical decision making for cancer treatment [109], many more institutions and clinicians could have access to this information. Expanding this technology to mobile phones, as with CNNs discussed above, could allow even greater accessibility [67], but the scalability of these methods to settings with limited resources remains to be demonstrated. 

Predicting MSI and other molecular features from H&E slides is an attractive goal given the success of CNNs in similar tasks and initial encouraging results. The focus of the published literature has been on H&E stained slides. It is possible that performance can be further improved by using additional histological stains. The previous performance of models created by human pathologists may also point to the attainability of this goal. The most important hurdle will be to demonstrate, through rigorous clinical trials, that utilizing machine learning on clinical samples is superior or non-inferior to the standard of care, which is itself rapidly evolving and non-uniform. For example, next generation sequencing is increasingly performed on tumor specimens, permitting the identification of MSI as part of a broader, clinically relevant, molecular characterization [110,111]. With decreasing sequencing costs and the need to detect certain mutations clinically (e.g., in *KRAS* and *BRAF)*, accurate genomic predictions in addition to MSI classification may be required from histology-based machine learning methods. Blood based tests have shown good accuracy for predicting MSI from the primary tumor [112] and radiomics have also been proposed for the prediction of MSI status [113,114,115]. 

Another major challenge is the generalizability of these methods, which, contrary to molecular methods such as PCR or immunohistochemistry, are often not robust to differing patient or tissue characteristics. The reduction in performance seen in several studies when a trained CNN was applied to new datasets may be a barrier to the widespread implementation of these methods (Table 3). 

The few peer-reviewed data that are available suggest that the accuracy of the current machine learning algorithms for the prediction of MSI may not yet be sufficient to guide clinical care in high-resource settings. However, as methods continue to improve and more training datasets become available, it is plausible that CNNs will be able to predict MSI status more accurately. Predictions for differing populations, primary cancer sites and tissue preparation methods (including true biopsies) are some of the challenges that exist. The accuracy of prediction for post-treatment pathology slides has not been explored and may be relevant for rectal cancer patients undergoing neoadjuvant therapy. Until the performance of CNNs improves, emphasis could be placed on achieving a near-perfect sensitivity for the detection of MSI, tolerating a certain number of false positives. MSI/dMMR assays could be avoided for most samples, but confirmation of CNN-predicted MSI would be required. Since MSI is a biomarker, the major potential for machine learning to improve upon MSI testing is if it were able to predict clinically relevant genomic features, such as *MLH1* hypermethylation or germline dMMR mutations, or clinically relevant outcomes, such as response to chemotherapy and immunotherapy (Table 3). The Immunoscore already uses digital pathology for prognostication [10,57] but machine learning could be utilized to improve predictions and to identify subsets of patients with microsatellite stable CRC that could benefit from immunotherapy [81].

Another limitation of the current research is that, to our knowledge, all CNN models trained to identify MSI status in CRC have been trained on surgical samples derived from resection of the primary tumor. Under current guidelines, immunotherapy is most commonly used to treat patients with stage IV tumors, who often have tissue samples available only from biopsies of metastatic sites. If machine learning models are not able to accurately predict MSI based on such samples, one of the most promising applications of MSI prediction from histological samples would be restricted to a much smaller segment of potential beneficiaries. Thus, future research should work to optimize machine learning algorithms for the prediction of MSI from biopsy samples of distant metastases.

Lastly, to accelerate the acceptance of CNNs as clinical tools and inform other areas of research, further insights into the features that drive CNN classification are needed. Without understanding what features CNNs are using to classify images, there exists a risk of introducing bias and error.

## Figures and Tables

**Table 1:** Histological predictors of microsatellite instability.

**A**					
**Variable**		**Sensitivity % (95% CI)**	**Specificity % (95% CI)**	**Odds Ratio, Univariate (95% CI)**	**Odds Ratio, Multivariate (95% CI)**
**Host response features**					
Tumor-infiltrating lymphocytes (TILs)		70 [26]	76 [26]	7.4 (5.4–10.3) [26]	3.8 (2.5–5.6) [26]
		72 (64–78) [27]	82 (80–85) [27]		9.1 (5.9–14.1) [27]
		21 [30]	97 [30]	9.8 (3.5–28.5) [30]	
		60 (50–70) [36]	78 (76–79) [36]	5.2 (3.2–8.5) [36]	3.7 (2.0–6.8) [36]
Crohn’s-like lymphocytic reaction (CLR)		68 [26]	54 [26]	2.5 (1.8–3.4) [26]	2.3 (1.6–3.5) [26]
		56 (48–63) [27]	77 (74–80) [27]		1.9 (1.2–2.9) [27]
		49 [30]	64 [30]	1.7 (0.9–3.2) [30]	
		69 (59–78) [36]	45 (44–46) [36]	1.8 (1.1–3.0) [36]	1.1 (0.6–2.0) [36]
Peritumoral lymphocytic reaction		86 (77–92) [36]	42 (40–42) [36]	4.3 (2.3–8.3) [36]	3.7 (1.6–8.6) [36]
Stromal plasma cells		78 (68–86) [36]	48 (46–48) [36]	3.2 (1.9–5.6) [36]	2.1 (1.1–4.1) [36]
**Tumor characteristics**					
Mucinous morphology *		53 [26]	80 [26]	4.6 (3.4–6.3) [26]	1.7 (1.1–2.7) [26]
		28 (21–35) [27] **	91 (89–93) [27] **		2.8 (1.7–4.8) [27] **
		22 [30]	93 [30]	3.7 (1.7–8.0) [30]	
		51 (41–61) [36]	78 (77–79) [36]	3.7 (2.3–6.0) [36]	4.71 (2.1–10.7) [36]
					2.13 (1.3–3.4) [37]
Medullary morphology (10–70%)		25 [30]	97 [30]	12.5 (4.6–35.9) [30]	
Grade †		64 [26] †	81 [26] †	7.4 (5.4–10.1) [26] †	3.4 (2.2–5.2) [26] †
		38 (31–46) [27]	82 (79–84) [27]		1.9 (1.2–3.1) [27]
		38 [30]	87 [30]	4.0 (2.2–7.3) [30]	
		17 (10–26) [36]	90 (89–91) [36]	1.8 (0.9–3.5) [36]	
		32 (23–43) [36] †	77 (76–78) [36] †	1.6 (0.9–2.6) [36] †	
Signet ring cells				4.3 (2.2–8.7) [26]	
		13 [30]	95 [30]	2.7 (1.1–6.8) [30]	
Lack of dirty or garland necrosis		59 [26]	79 [26]	5.4 (3.9–7.4) [26]	1.8 (1.1–2.8) [26]
		26 (18–35) [36]	89 (88–90) [36]	2.7 (1.5–4.7) [36]	1.4 (0.7–3.0) [36]
Cribriform pattern		13 [30]	72 [30]	0.4 (0.2–0.8) [30]	
Histologic heterogeneity				4.4 (3.0–6.4) [26]	
		55 (45–65) [36]	69 (68–70) [36]	2.7 (1.7–4.4) [36]	
**Clinical/Molecular Features**					
Age <50 years				2.2 (1.3–3.8) [26]	3.1 (1.5–6.5) [26]
		52 (44–60) [27]	59 (56–62) [27]		1.9 (1.3–2.9) [27]
		21 (13–29) [36]	89 (88–90) [36]	2.0 (1.1–3.7) [36]	3.8 (1.8–8.0) [36]
Female				1.4 (1.0–1.9) [26]	1.3 (0.7–2.2) [36]
		51 (41–62) [36]	63 (62–64) [36]	1.8 (1.1–2.8) [36]	1.56 (1.0–2.4) [37]
Size > or equal to 60 mm					2.75 (1.8–4.2) [37]
Anatomic site (right sided/proximal)		70 [26]	63 [26]	4.1 (2.9–5.7) [26]	2.2 (1.5–3.3) [26]
		74 (67–81) [27]	70 (67–73) [27]		4.7 (3.1–7.3) [27]
		79 (70–87) [36]	63 (61–63) [36]	6.4 (3.6–11.2) [36]	5.08 (2.7–9.6) [36]
					3.76 (2.4–5.9) [37]
*BRAF* mutant					13.33 (8.0–22.2) [37]
**B**					
**Model**	**Model Variables**	**Sensitivity (%)**	**Specificity (%)**	**Positive/Negative Predictive Value (%)**	**AUC or Accuracy (95% CI)**
Greenson et al. ** [26]	TIL/HPF, well or poorly differentiated, age < 50, CLR, R-sided, lack of dirty necrosis, any mucinous differentiation	92 **	46 **		AUC 0.850 **
MsPath [27]	Age < 50, proximal location, mucinous/signet ring/undifferentiated, poorly differentiated, CLR, TILs	93	55		AUC 0.890 (0.83– 0.94)
PREDICT [36]	R-sided, mucinous component, age < 50 years, TILs, peritumoral reaction, increased stromal plasma cells	96.9	76.6	35.2/99.5	AUC 0.924
Fujiyoshi et al. [37]	Female, mucinous component, tumor size > or equal to 60 mm, proximal location, *BRAF* mutation	76	77		AUC 0.856 (0.806 –0.905)
RERTest6 ** [38]	Proximal location, expansive growth pattern, CLR, solid pattern %, mucinous pattern %, cribriform pattern	78.01 *	93.39 **	51.8/97.9 **	Accuracy 0.921 **

Table 1 summarizes individual features and composite models used for the prediction of microsatellite instability (MSI) from histology. (A) Individual variables associated with MSI are listed. * Jenkins et al. [27] included signet cell and medullary morphology with mucinous morphology. † These studies included either poorly or well differentiated tumors as associated with MSI. (B) Multivariable models and their performance in differentiating between tumors with and without MSI are presented. ** Values are presented for validation cohorts when available. Greenson et al. and RERTest6 did not include a separate validation cohort. CI: confidence interval. TIL: tumor infiltrating lymphocytes. CLR: Crohn’s-like lymphoid reaction. HPF: high power field. R-sided: right sided. AUC: area under the curve.

**Table 2:** Deep learning for prediction of MSI from digital pathology.

CNN and Additional Methods	Other CNNs Evaluated	Training Cohort	Test Cohort(s) with AUC (95% CI) or Accuracy	External Validation Cohort(s) with AUC (95% CI)
ResNet-18 Whole-slide image classified per majority of image tiles [43]	AlexNet, VGG-19, InceptionV3, SqueezeNet	TCGA CRC FFPE	TGCA CRC FFPEAUC 0.77 (0.62–0.87)	DACHS CRC FFPE AUC 0.84 (0.72–0.92)
		TCGA CRC frozen	TCGA CRC frozen AUC 0.84 (0.73–0.91)	DACHS CRC FFPE 0.61 (0.50–0.73)
		TCGA gastric FFPE	TCGA gastric FFPE AUC 0.81 (0.69–0.90)	DACHS CRC FFPE AUC 0.60 (0.48–0.69) KCCH gastric FFPE AUC 0.69 (0.52–0.82)
		TCGA uterine FFPE	TCGA uterine FFPE AUC 0.75 (0.63–0.83)	
ShuffleNet [67]	AlexNet, InceptionV3, ResNet-18, DenseNet201	TCGA CRC	TCGA CRC AUC 0.805	DACHS CRC AUC 0.89 (0.88–0.92)
ResNet-18 Whole slide image classified using two multiple instance learning pipelines integrated into an ensemble classifier [68]	none	TCGA CRC frozen	TCGA CRC frozen AUC 0.885	Asian CRC AUC FFPE 0.650
		TCGA CRC frozen with 10% Asian CRC FFPE	Asian CRC FFPEAUC 0.850	
		TCGA CRC frozen with 70% Asian CRC FFPE	Asian CRC FFPEAUC 0.926	
Custom multilayer perceptron (HE2RNA) applied after feature extraction by ResNet-50; with and without transcriptomic representation of histology [69]	none	TCGA CRC FFPE, with transcriptomic representation and 20% of training cohort	TCGA CRC FFPEAUC ~0.80 *	
		TCGA CRC FFPE, using >80% of training cohort	TCGA CRC FFPEAUC ~0.80 *	
Inception-V3 with and without adversarial learning [70]	VGG-19, ResNet-50	TCGA CRC	TCGA CRC Accuracy 98.3%	TCGA endometrial Accuracy 53.7%
		TCGA CRC and endometrial	TCGA CRC Accuracy 72.3% TCGA endometrial Accuracy 84.2%	TCGA gastric Accuracy 34.9%
		TCGA CRC and endometrial with adversarial learning	TCGA CRC Accuracy 85.0%TCGA endometrial Accuracy 94.6%	TCGA gastric Accuracy 57.4%
InceptionResNetV1 [71]	InceptionV1-3, InceptionResnetV1-2, Panoptes1-4 (multibranch custom InceptionResnet)	TCGA and CPTAC endometrial carcinoma	TCGA and CPTAC endometrial carcinoma AUC 0.827 (0.705–0.948)	
ShuffleNet [72]	none	MSIDETECT CRC (color normalized)	MSIDETECT CRC AUC 0.92 (0.90–0.93)	YCR-BCIP CRC surgical samples AUC 0.96 (0.93–0.98)
				YCR-BCIP CRC biopsy samples AUC 0.78 (0.75–0.81)
		YCR-BCIP CRC biopsy samples	YCR-BCIP CRC biopsy samples	

Table 2 summarizes the methods and performance of available data regarding deep learning for MSI prediction from pathology. * Exact AUC not reported, estimate based on an included graphical representation. CNN: convolutional neural network. TCGA: The Cancer Genome Atlas, CRC: colorectal cancer, DACHS: CRC prevention through screening study (abbreviation in German), FFPE: Formalin-Fixed Paraffin-Embedded, AUC: Area under the receiver operating characteristic curve, KCCH: Kanagawa Cancer Center Hospital (Japan), CPTAC: Clinical Proteomic Tumor Analysis Consortium, MSIDETECT: A consortium comprised of whole slide images from TCGA, DACHS, the United Kingdom-based Quick and Simple and Reliable trial (QUASAR), and the Netherlands Cohort Study (NLCS), YCR-BCIP: Yorkshire Cancer Research Bowel Cancer Improvement Programme.

**Table 3:** Advantages, limitations, and future directions for MSI classification from digital pathology using machine learning.

	Advantages	Limitations	Future Directions
Classification accuracy	- Rapidly improving accuracy of several CNN architectures	- Insufficient accuracy to obviate universal MSI screening in resource-rich settings currently - Decreased accuracy when applied to biopsy samples - Lacking data on post treatment samples (e.g., rectal cancer)	- Determine optimal CNN architecture/s for distinct clinical scenarios - Leverage machine learning to prioritize molecular testing in settings with limited resources
Generalizability	Excellent performance on well curated cohorts that are similar to training data	Performance not robust to differing patient and tissue characteristics	Increase availability of datasets for global and local model refinement
Accessibility	Potential to expand access to settings without pathology experts or molecular testing, including via cellular devices	- Digital pathology not yet widely available - No published studies conducted in settings with reduced access to healthcare	Design dedicated CNNs for settings with reduced access to healthcare
Clinical endpoint prediction	Very good classification of MSI	No direct prediction of clinical endpoints	Shift from surrogate marker classification to clinical endpoint prediction
Patient selection for immunotherapy		Decreased accuracy of MSI classification in metastatic disease, where immunotherapy is approved	- Directly predict response to immunotherapy, regardless of MSI status - Improve MSI prediction in metastatic disease
Identification of Lynch Syndrome		Inability to distinguish between somatic and germline etiology of MSI, such that confirmatory testing is required	- Predict somatic vs. germline etiology of MSI
Comparison with next generation sequencing (NGS)	Rapid and cost-effective after initial investment	Cannot currently reliably detect *KRAS* or *BRAF* mutations, tumor mutational burden and other clinically actionable alterations	- Attempt to directly predict response to therapy based on histology - Improve classification of other molecular alterations - Integrate genomic and histologic data for optimal prediction
Cost effectiveness	Long term savings on molecular assays	Initial investment required in hardware and software for digital pathology	Expand use of and access to digital pathology

Table 3 outlines the advantages, limitations, and future directions as they relate to detecting MSI from histology using machine learning. CNN: convolutional neural network.

## Data Availability

All the data used for this review is available by accessing the citations below.
